# Insulin‐like growth factor binding protein‐4 exerts antifibrotic activity by reducing levels of connective tissue growth factor and the C‐X‐C chemokine receptor 4

**DOI:** 10.1096/fba.2018-00015

**Published:** 2019-01-15

**Authors:** YunYun Su, Tetsuya Nishimoto, Stanley Hoffman, Xinh‐Xinh Nguyen, Joseph M. Pilewski, Carol Feghali‐Bostwick

**Affiliations:** ^1^ Division of Rheumatology and Clinical Immunology, Department of Medicine Medical University of South Carolina Charleston South Carolina; ^2^ Division of Pulmonary, Allergy, and Critical Care, Department of Medicine University of Pittsburgh Pittsburgh Pennsylvania

**Keywords:** extracellular matrix (ECM), fibroblast, pulmonary fibrosis, systemic sclerosis (SSc)

## Abstract

The insulin‐like growth factor (IGF) system plays an important role in variety cellular biological functions; we previously reported levels of IGF binding proteins (IGFBP)‐3 and ‐5 are increased in dermal and pulmonary fibrosis associated with the prototypic fibrosing disease systemic sclerosis (SSc), induce extracellular matrix (ECM) production, and promote fibrosis. We sought to examine the effects of another member of the family, IGFBP‐4, on ECM production and fibrosis using cell‐based, ex vivo organ culture and in vivo mouse lung fibrosis models. IGFBP‐4 mRNA levels were significantly decreased in pulmonary fibroblasts of patients with SSc. ECM components were significantly reduced by endogenous and exogenous IGFBP‐4. IGFBP‐4 also blocked TGF‐β–induced ECM production, and inhibited ECM production ex vivo in human lung and skin in organ culture. In vivo, IGFBP‐4 reduced bleomycin‐induced collagen production and histologic evidence of fibrosis. Silencing IGFBP‐4 expression to mimic levels observed in SSc lung fibroblasts resulted in increased ECM production. IGFBP‐4 reduced mRNA and protein levels of the chemokine receptor CXCR4 and the profibrotic factor CTGF. Furthermore, CTGF silencing potentiated the antifibrotic effects of IGFBP‐4. Reduced IGFBP‐4 levels in SSc lung fibroblasts may contribute to the fibrotic phenotype via loss of IGFBP‐4 antifibrotic activity.

**Abbreviations**
BAL: bronchoalveolar lavage
CTGF: connective tissue growth factor
CXCR4: C‐X‐C chemokine receptor 4
ECM: extracellular matrix
FN: fibronectin
IGF: insulin‐like growth factor
IGFBP‐4(3,5): insulin‐like growth factor binding protein‐4(3,5)
INF‐β: interferon‐beta
IPF: idiopathic pulmonary fibrosis
MOI: multiplicity of infection
PDGF: platelet‐derived growth factor
rhIGFBP‐4: recombinant human IGFBP‐4
SDF‐1: stromal cell‐derived factor‐1
siRNA: small‐interfering RNA
SSc: systemic sclerosis
TGF‐β: transforming growth factor‐beta
TβRI(II): TGF‐ß receptor (types I‐II)
VEGF: vascular endothelial growth factor
WB: western blot
α‐SMA: alpha‐smooth muscle actin

## 1 INTRODUCTION

Systemic sclerosis (SSc) is a connective tissue disease characterized by vascular injury, immune system abnormalities, and organ fibrosis. Lung involvement is currently the leading cause of death in patients with SSc^1^ and the only therapeutic option is lung transplantation. However, the scarcity of donor lungs and high cost of transplantation restrict its wide application. Thus, identifying new antifibrotic agents for clinical use is critically needed.

Pulmonary fibrosis in SSc and related disorders is characterized by excessive extracellular matrix (ECM) deposition in lung parenchyma leading to distortion of pulmonary architecture and loss of function. Profibrotic factors in the lung milieu lead to activation of cells, including fibroblasts, and their transition to myofibroblasts which have characteristics of smooth muscle cells, a more contractile phenotype, and increased expression of alpha‐smooth muscle actin (α‐SMA) and ECM proteins.^2^, ^3^

Insulin‐like growth binding proteins (IGFBP) were originally described as insulin‐like growth factor (IGF) transport proteins, potentiating or inhibiting IGF function. However, IGF‐independent functions of IGFBPs have emerged as some of their most important functions. We previously reported that two members of the IGFBP family of proteins, IGFBP‐3 and IGFBP‐5, induce ECM production and deposition and promote fibrosis in an IGF‐independent manner.^4^, ^5^, ^6^ We further reported that IGFBP‐5 induced pulmonary and dermal fibrosis in vivo and both proteins induced dermal fibrosis ex vivo in human skin maintained in organ culture^7^, ^8^. Furthermore, IGFBP‐5 exerted chemotactic activity and triggered epithelial to mesenchymal transition.^7^, ^9^ In contrast to IGFBP‐3 and IGFBP‐5, IGFBP‐4 did not promote fibrosis in our ex vivo skin model.^10^ The reported activities of IGFBP‐4 have included its antiangiogenic and antitumorigenic activity in endothelial cells and glioblastoma cells,^11^ respectively, which have been shown to be IGF independent.^12^ In addition, Gimbel et al reported that intraperitoneal treatment of rats with IGFBP‐4 reduced tissue adhesions after abdominal surgery.^13^ We therefore sought to examine the effects of IGFBP‐4 on ECM production and fibrosis in our in vitro, in vivo, and ex vivo models of pulmonary fibrosis. Our findings demonstrate that IGFBP‐4 exerts antifibrotic effects in vitro, ex vivo, and in vivo, and its levels are reduced in primary pulmonary fibroblasts of patients with SSc‐associated pulmonary fibrosis. Furthermore, the antifibrotic effects of IGFBP‐4 are mediated via downregulation of the chemokine stromal cell‐derived factor (SDF‐1) receptor C‐X‐C chemokine receptor 4 (CXCR4) and inhibition of connective tissue growth factor (CTGF).

## 2 MATERIALS AND METHODS

### 2.1 Human lung fibroblast culture

Primary human lung fibroblasts were cultured from normal donor lungs as previously described^4^ and used in passages 4‐7. Fibroblasts were also cultured from lung tissues of patients with SSc‐associated pulmonary fibrosis who underwent lung transplantation at the University of Pittsburgh Medical Center as previously described.^14^ MRC‐5 cell line was purchased from American Type Culture Collection (ATCC, Manassas, VA). Cells were grown in Dulbecco's Modified Eagle Medium (DMEM) (Mediatech, Inc Manassas, VA) supplemented with 10% fetal bovine serum (FBS). Fibroblasts were cultured in serum‐free medium overnight prior to treatment with recombinant human IGFBP‐4 (rhIGFBP‐4) (ThermoFisher Scientific, Waltham, MA) or transforming growth factor‐beta1 (TGF‐ß1) (R&D Systems, Minneapolis, MN) at 2 µg/mL and 10 ng/mL, respectively. Following treatment, fibroblasts were maintained in DMEM with 0.5% FBS for the indicated times. For endogenous expression of IGFBP‐4, fibroblasts were infected with replication‐deficient adenovirus encoding IGFBP‐4 or a control vector at a multiplicity of infection (MOI) of 50 as we previously described.^4^

### 2.2 Generation of IGFBP‐4‐expressing constructs

The full‐length human IGFBP‐4 cDNA was generated from normal lung fibroblasts, amplified with forward primer 5′‐AAAGGATCCATGCTGCCCCTCTGCCTCGT‐3′, and 6X his tag‐containing reverse primer 5′‐AAAGAATTCTCAATGATGATGATGATGATGCTCTCGAAAGCTGTCAG‐3′, digested with BamH1 and EcoR1, and cloned into the shuttle vector pAdlox. IGFBP‐4 with a mutated IGF‐binding site was generated from our IGFBP‐4 cDNA template by mutating amino acid residues 74‐75 from histidine (H) and glycine (G) to proline (P) and threonine (T)^15^ by a commercial source (GeneWiz, South Plainfield, NJ, USA). The sequence of each construct was confirmed and the corresponding plasmids were purified using a Plasmid Maxi kit (Qiagen, Valencia, CA). HEK293T cells were transfected using Lipofectamine 3000 (Invitrogen, Grand Island, NY) and the supernatants were harvested for purification of wild‐type and mutant IGFBP‐4. Adenoviruses expressing the wild‐type or mutant IGFBP‐4 sequences were generated in the Vector Core Facility of the University of Pittsburgh as previously described.^4^

### 2.3 IGFBP‐4 protein purification

Conditioned medium was prepared by culturing HEK293T cells transfected with pAdlox expressing His‐tagged wild‐type or mutant IGFBP‐4 in Opti‐MEM® I Reduced‐Serum Medium (Life Technologies, Grand Island, NY) or DMEM supplemented with 2% FBS. The medium was supplemented with 1/9th volume 1 M Tris (pH 8.0), 1/20th volume 3 M NaCl, and 1/500th volume 5 M imidazole. In a typical preparation, 50 mL of adjusted medium was then incubated with 1 mL HisPur Cobalt Resin (ThermoFisher, Scientific, Waltham, MA) by rotation at room temperature for 2 hours. The beads were then collected by serial low‐speed centrifugation and washed six times by dilution with 10 mL 0.1 M Tris, pH 8.0/0.3 M NaCl (TN). The beads were then transferred to a 10‐mL disposable column, washed once with 10 mL TN, and eluted with TN/250 mM imidazole (0.7 mL, 1 mL, 1 mL). Essentially all of the purified IGFBP‐4 was in the first 1 mL eluate. To transfer this IGFBP‐4 into phosphate‐buffered saline (PBS), the eluate was dialyzed three times vs 500 volumes of PBS.

### 2.4 Ex vivo human lung and skin culture

Normal human lung was obtained from donors whose lungs were not used for transplantation as previously described.^4^ Lung tissues were also obtained from patients with SSc‐associated pulmonary fibrosis who underwent lung transplantation at the University of Pittsburgh Medical Center as previously described.^14^ Lung tissue cores were generated using a 5‐mm punch. Lung cores were maintained in culture in a six‐well plate containing 3 cores per well and 1.5 mL of DMEM/10% FBS. Adenoviruses were added at 1.5 × 10^8^ pfu/well. Some wells were treated with 2 µg/mL rhIGFBP‐4 with or without 10 ng/mL TGF‐ß1 for 4 or 7 days. Lung cores were snap frozen and homogenized either in QIAzol lysis reagent (Qiagen, Valencia, CA) for RNA extraction, or 10 mM Tris buffer pH 8.0 with protease inhibitor cocktail (Sigma, St Louis, MO) for total protein extraction. Total lung RNA was purified using the RNeasy microarray tissue mini kit (Qiagen) following the manufacturer instructions. Human newborn foreskin was obtained from the nursery at the Medical University of South Carolina (MUSC). Skin punches, 3 mm each, were prepared and four punches were cultured in each well of a six‐well culture dish. Skin was maintained in organ culture with the dermis immersed in medium while the epidermis remained exposed to air. Following treatment with recombinant proteins or adenoviruses, supernatants were collected and skin pieces were snap frozen for the measurement of hydroxyproline levels.

### 2.5 In vivo mouse experiments

Six‐ to eight‐week‐old C57BL/6J mice were purchased from the Jackson Laboratory (Bar Harbor, Maine). Mouse experiments were approved by the MUSC IACUC. Pulmonary fibrosis was induced in mice following intratracheal administration of 1.2 mU/g bleomycin mixed with 10 µg rhIGFBP‐4 or vehicle in a volume of 50 µL. rhIGFBP‐4 or mutant IGFBP‐4 were administered to different mice on days 0, 3, and 6 post‐bleomycin for a total of three doses. On day 21, mice were euthanized and lungs were gently perfused with 1X PBS. The left lung was collected for hydroxyproline assay. The right lung was further perfused with 10% zinc formalin fixative (Polysciences Inc, Warrington, PA) and fixed in 10% zinc formalin for 48 hours prior to paraffin embedding and histological analysis.

### 2.6 Hydroxyproline assay

Hydroxyproline content in lung and skin tissues was measured as previously described.^16^

### 2.7 Real‐time PCR and PCR array

Total RNA was isolated from primary fibroblasts and lung tissues using the RNeasy mini kit and RNeasy microarray tissue mini kit (Qiagen), respectively. cDNA was synthesized using SuperScript ǁ reverse transcriptase (ThermoFisher Scientific). Real‐time PCR was carried out using commercial primer TaqMan probes with FAM or VIC label for CXCR4, collagen 1A2, fibronectin, IGFBP‐4, CTGF, α‐SMA, and glyceraldehyde 3‐phosphate dehydrogenase (GAPDH), in Master Mix reagent (ThermoFisher Scientific) in an ABI Prism 7300 or a StepOnePlus™ Real‐Time PCR System (ABI, Applied Biosystems, Carlsbad, CA, USA). Gene expression levels were calculated by using the ^ΔΔ^Ct method, and signals were normalized to levels of the housekeeping gene GAPDH. Gene expression analysis was also examined using the human fibrosis PCR Array (PAHS‐120Z) kit (SABiosciences, Frederick, MD) according to the manufacturer's instructions. Data were analyzed using the SABiosiences Web‐Based PCR Array data analysis system.

### 2.8 Small‐interfering RNA (siRNA) transfection

Primary human lung fibroblasts were seeded in six‐well plates 24‐48 hours prior to transfection with siRNA. IGFBP‐4 and CTGF sequence‐specific siRNA and negative control scrambled siRNA were purchased from Applied Biosystems/Ambion (Austin, TX). For transfection, Lipofectamine 2000 (Invitrogen, Grand Island, NY) and 100 nM siRNA diluted in Opti‐MEM® I Reduced‐Serum Medium (Life Technologies) were added to fibroblasts in culture following the manufacturer's recommendations. After 24 hours, transfected fibroblasts were treated with rhIGFBP‐4 in DMEM supplemented with 0.5% FBS.

### 2.9 Western blot (WB)

Whole cell lysates were prepared by scraping cells directly in 2× sodium dodecyl sulfate (SDS) sample buffer. In parallel experiments, membrane proteins were extracted using the Subcellular Protein Fractionation kit (Pierce Biotechnology, Rockford, IL) following the manufacturer's instructions. Extracellular matrix was prepared as we previously described.^4^ Proteins were separated by SDS‐polyacrylamide gel electrophoresis (SDS‐PAGE) and transferred to PVDF membranes. Membranes were blocked with 5% nonfat dry milk in Tris‐buffered saline (TBS)/0.05% Tween‐20 (TBST) buffer then incubated with antibodies against human IGFBP‐4, collagen, fibronectin, tenascin‐C, CTGF, GAPDH (Santa Cruz, Dallas, TX), phosphorylated SMAD2 and SMAD3, total SMAD2 and SMAD3, phosphorylated SMAD1/5/9, phosphorylated (p)‐44/42 MAPK, p‐AKT, p‐P38 kinase, p‐SAPK/JUNK, and antibodies targeting the corresponding total proteins (Cell Signaling Tech, Danvers, MA), CXCR4 and α‐SMA (Abcam, Cambridge, MA), His tag (Sigma), or tubulin (Epitomics Inc, Burlingame, CA), washed with TBS three times, and then incubated with horseradish peroxidase‐labeled secondary antibody (Santa Cruz). Signals were detected by chemiluminescence (Perkin Elmer, Waltham, MA, USA) on a FluorChem R system (ProteinSimple, San Jose, CA). Images were analyzed using Image J.

### 2.10 Hematoxylin and eosin, immunofluorescence, and immunohistochemistry

Following fixation in 10% zinc formalin (Polysciences Inc) for 48 hours, mouse right lungs were embedded in paraffin and sectioned. Sections were stained with hematoxylin and eosin (H&E) and histological images captured on a Moticam Pro BA410 microscope (Speed Fair Co., Ltd, Hong Kong, China). α‐SMA was detected in mouse lung sections using immunofluorescence. Briefly, following deparaffinization in xylene and rehydration in graded ethanol baths, antigen retrieval of paraffin‐embedded sections was done in sodium citrate buffer (10 mM sodium citrate, 0.05% Tween 20, pH 6.0) in a 95‐100°C steam bath for 20 minutes. Endogenous biotin was blocked using Avidin/Biotin blocking solution (Vector Labs, Burlingame, CA). Sections were incubated with normal goat serum blocking solution (2% goat serum, 1% BSA, 0.1% Triton‐100, 0.05% Tween20, 0.01 M PBS, pH 7.2) for blocking nonspecific background and permeabilization. Sections were then incubated overnight at 4°C with rabbit anti‐α‐SMA (Abcam), washed three times with 1X PBS, incubated with biotinylated secondary antibody for 1 hour at room temperature, and then conjugated with Fluorescein Avidin DCS (Vector Labs) for 20 minutes. Hoechst (Sigma) was used to identify nuclei. Images were captured on a Moticam Pro BA410 microscope (Speed Fair Co., Ltd, Hong Kong, China). For immunohistochemistry, the sections were quenched with BLOXALL Endogenous Peroxidase and Alkaline Phosphatase Blocking Solution (Vector Labs) for 15 minutes followed by incubation first with rabbit anti‐α‐SMA antibody (Abcam) at 1:500 dilution at 4°C for overnight. Sections were then washed with PBS/0.05% Tween‐20 (PBST) buffer three times, and incubated with ImmPRESS HRP Anti‐Rabbit IgG (Vector Labs) for 1 hour at room temperature. The α‐SMA signal was developed by DAB substrate and counterstained by Gill's hematoxylin (Vector Labs). Images were captured by a Zeiss AxioObserver.Z1 (Carl Zeiss MicroImaging GmbH, 07740 Jena, Germany); blood vessel numbers were counted through at least 10 different 20x magnification fields from three different samples for each group.

### 2.11 Western ligand blot

Western ligand blotting was used to detect IGF binding activity as previously described.^4^ Briefly, 25 µg of purified IGFBP‐4 and mutant IGFBP‐4 were resolved on SDS‐PAGE under nonreducing conditions. Proteins were transferred to a PVDF membrane. The membrane was blocked with 5% nonfat dry milk at room temperature for 1 hour, incubated with 0.5 µg/mL biotinylated IGF‐I (GroPep, Thebarton, SA, Australia) in TBST at 4°C overnight, washed with TBST, and incubated with streptavidin‐HRP (Amersham, Piscataway, NJ, USA) for 1 hour at room temperature, and then washed. Signal was detected using Chemiluminescence (Perkin Elmer, Waltham, MA). Images were analyzed using Image J.

### 2.12 Statistical analysis

Statistical analysis of the data was performed using the Student's *t* test for two comparisons and ANOVA with post‐hoc Bonferroni for multiple comparisons. The significance level was set at *P* < 0.05. GraphPad Prism version 7 for Windows (GraphPad Software, La Jolla, CA) was used to analyze data.

## 3 RESULTS

### 3.1 IGFBP‐4 reduces baseline and TGF‐ß induced ECM production

**Figure 1:**
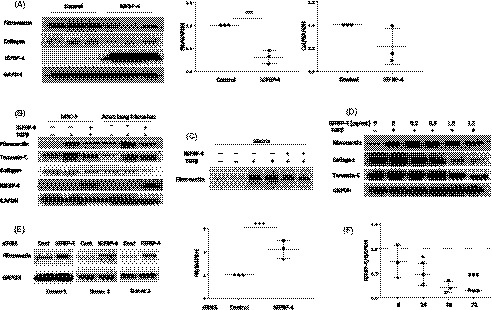
IGFBP‐4 reduces baseline and TGF‐ß–induced ECM production. (A) Endogenous adenovirally‐expressed IGFBP‐4 reduces ECM levels. Human adult lung fibroblasts were infected with replication‐deficient adenovirus encoding IGFBP‐4 or control adenovirus for 72 hours. Lysates were harvested and levels of collagen and fibronectin analyzed by western blot. Experiments were done in triplicate. Graphical presentation of the data is shown on the right. (B) Endogenous IGFBP‐4 reduces the TGF‐ß–induced ECM proteins fibronectin, collagen, and tenascin‐C in fetal lung and adult lung fibroblasts. MRC‐5 cells and primary human adult lung fibroblasts were infected with replication‐deficient adenovirus‐expressing IGFBP‐4 or control adenovirus for 24 hours and stimulated with 10 ng/mL TGF‐ß1 for an additional 48 hours. Cellular lysates were assessed for the indicated ECM proteins using western blot. The experiments were done three times with similar results. (C) Endogenous IGFBP‐4 reduces TGF‐ß–induced fibronectin in the matrix. Primary human adult fibroblasts were treated as in B and extracellular matrix fractions were harvested and analyzed by WB. The experiments were done three times, each time in duplicate, with similar results. (D) Exogenous IGFBP‐4 exerts similar antifibrotic effects in a dose‐dependent manner. Primary human adult lung fibroblasts were treated with 10 ng/mL TGF‐ß1 and the indicated concentrations of rhIGFBP‐4 for 72 hours. Cellular lysates were analyzed by WB. The experiments were done three times with similar results. (E). Silencing IGFBP‐4 increases fibronectin. Primary human lung fibroblasts were transfected with siRNA targeting IGFBP‐4 or control scrambled siRNA for 72 hours. Cellular lysates were analyzed by WB. Graphical presentation of the data is shown on the right. (F) TGF‐ß reduces IGFBP‐4 expression in a time‐dependent manner from 6 h to 72 h. *P < 0.05, **P < 0.01, ***P < 0.0001

To assess the effect of IGFBP‐4 on ECM production, we first tested its effects on untreated primary human adult lung fibroblasts. Fibroblasts were infected with a replication‐deficient adenovirus expressing human IGFBP‐4 or a control adenovirus. Our results show that IGFBP‐4 significantly reduced baseline levels of the ECM components fibronectin (FN) and collagen in cellular lysates (Figure 1A). IGFBP‐4 also inhibited TGF‐ß induced production of collagen, fibronectin, and tenascin‐C in MRC‐5 fibroblasts and adult human lung fibroblasts (Figure 1B). In addition to reducing ECM production in cellular lysates, IGFBP‐4 also reduced fibronectin levels in the ECM fraction (Figure 1C). Since endogenous and exogenous IGFBPs can exert different effects, we also tested the effect of exogenous rhIGFBP‐4. Exogenous rhIGFBP‐4 exerted similar effects to endogenously produced protein and its ECM‐lowering effect was dose‐dependent (Figure 1D). To further validate the effects of gain of function of IGFBP‐4 on ECM reduction, we examined the effect of loss of function of IGFBP‐4 in primary human lung fibroblasts. To do so, we silenced IGFBP‐4 using sequence‐specific siRNA. IGFBP‐4 deficiency in vitro resulted in significantly increased production of the ECM protein fibronectin, further confirming the role of IGFBP‐4 in modulation of ECM levels (Figure 1E). To identify the mechanism by which IGFBP‐4 reduces ECM levels in primary fibroblasts, we examined the effects of IGFBP‐4 on different signaling pathways at different time points. IGFBP‐4 modestly reduced TGF‐ß induced phosphorylation of SMAD‐2 and ‐3 (supplemental Figure S1), but had no effect on SMAD‐1, ‐5, or ‐9 phosphorylation (data not shown). IGFBP‐4 also had no effect on the phosphorylation of p44/42 MAPK, AKT, SAPK/JNK, or P38 kinase (data not shown). These findings suggest that IGFBP‐4 mediated reduction of ECM levels likely occurs via modulation of the canonical TGF‐ß signaling pathway rather than the noncanonical TGF‐ß signaling pathway. Since TGF‐ß is the most potent profibrotic factor used experimentally, we also examined the effect of TGF‐ß on IGFBP‐4 expression. TGF‐ß significantly reduced expression of IGFBP‐4 in a time‐dependent manner (Figure 1F). Treatment of primary human lung fibroblasts with physiological concentrations of the profibrotic factors IGFBP‐3 and IGFBP‐5 did not reduce IGFBP‐4 expression (data not shown).

### 3.2 IGFBP‐4 decreases TGF‐ß induced ECM production ex vivo in human lung and skin.

**Figure 2:**
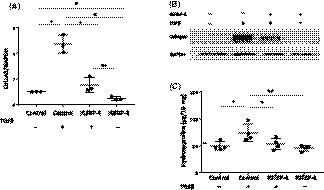
IGFBP‐4 decreases ECM production levels ex vivo in human lung and skin organ culture. (A) IGFBP‐4 decreases baseline and TGF‐ß– induced collagen mRNA levels in human lung. Normal human donor lung tissues were cultured with 2 µg/mL of rhIGFBP‐4 with or without 10 ng/mL TGF‐ß1 for 4 days. Collagen mRNA expression was analyzed by real‐time PCR. The graph shows results from lung tissues of three different donors. (B) IGFBP‐4 decreases baseline and TGF‐ß–induced collagen levels in human lung. Donor lungs were cultured as in A, except that the culture time was extended to 7 days. Lung tissues were homogenized and equal amounts of total protein analyzed by WB. The graph shows results from lung tissues of three different donors. (C) IGFBP‐4 decreases TGF‐ß–induced hydroxyproline levels in human skin. Human newborn foreskin was maintained in organ culture in the presence of 2 µg/mL rhIGFBP‐4 with or without 10 ng/mL TGF‐ß1 for 7 days. Collagen content was measured using a hydroxyproline assay. The graph includes results from the skin of four different donors. *P < 0.05, **P < 0.01

The ex vivo organ culture model allows the testing of pro‐ and antifibrotic factors in a human tissue, thus confirming the applicability of findings to humans and human diseases. We have optimized the use of human skin^10^, ^17^ and human lung (unpublished) for ex vivo testing. We first examined the effect of IGFBP‐4 in lung tissues. Human normal donor lungs were prepared as described in the Methods section and treated with rhIGFBP‐4 for 4 days (for RNA extraction) or 7 days (for protein extraction). Our results show that IGFBP‐4 reduces TGF‐ß–induced collagen 1A2 mRNA (Figure 2A) and protein levels (Figure 2B). We then extended our model to human skin. Human skin was maintained in organ culture and treated with rhIGFBP‐4 for 7 days. IGFBP‐4 reduced total collagen content in human skin as measured using hydroxyproline assay (Figure 2C).

### 3.3 IGFBP‐4 levels are decreased in SSc lung fibroblasts and IGFBP‐4 reduces existing fibrosis in SSc lungs

**Figure 3:**
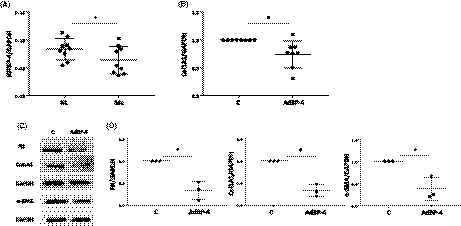
IGFBP‐4 expression is reduced in SSc lung fibroblasts and IGFBP‐4 decreases ECM and α‐SMA levels in SSc lung tissues in organ culture. (A) IGFBP‐4 expression is reduced in SSc fibroblasts. IGFBP‐4 mRNA was measured by real‐time PCR in primary pulmonary fibroblasts from nine patients with SSc and nine normal donors. All fibroblasts were in passage 3. Levels were normalized to GAPDH. (B) IGFBP‐4 reduces SSc lung collagen mRNA levels in ex vivo lung culture. Human SSc lung tissue cores were infected with adenovirus encoding IGFBP‐4 or control virus for 4 days. Collagen mRNA levels were measured by real‐time PCR. The graph shows results from eight different human SSc lung donors. (C) IGFBP‐4 reduces ECM production and levels of the myofibroblast marker α‐SMA. Human SSc lung tissues in ex vivo culture were infected with adenovirus encoding IGFBP‐4 or control virus for 7 days. Lung tissues were harvested and homogenized. An equal amount of total protein was analyzed by WB for collagen, fibronectin, and α‐SMA. GAPDH was used to confirm equal loading of protein. D. Graphical presentation of densitometry results from three different human SSc donor lung tissues. *P < 0.05

Since our data demonstrate IGFBP‐4 has antifibrotic activity, we measured IGFBP‐4 mRNA levels in primary fibroblasts from lung tissues of patients with SSc‐associated pulmonary fibrosis. IGFBP‐4 mRNA levels were significantly lower in SSc fibroblasts compared to control lung fibroblasts (Figure 3A). To further extend our findings and examine the ability of IGFBP‐4 to reduce ongoing severe fibrosis, we tested the effects of expressing human IGFBP‐4 in lung tissues derived from patients with SSc undergoing lung transplantation. IGFBP‐4 significantly reduced the mRNA levels of collagen 1A1 in SSc lung tissues maintained in organ culture (Figure 3B). Furthermore, IGFBP‐4 significantly reduced protein levels of the ECM proteins collagen and fibronectin as well as the myofibroblast marker α‐SMA in SSc lung tissue homogenates (Figure 3C and D).

### 3.4 IGFBP‐4 reduces pulmonary fibrosis in the murine bleomycin model in an IGF‐independent manner

**Figure 4:**
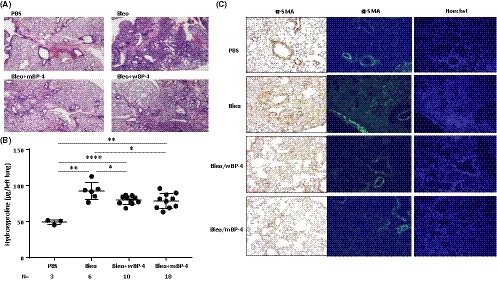
IGFBP‐4 attenuates bleomycin‐induced lung fibrosis and α‐SMA levels in vivo independently of IGF‐1. (A) IGFBP‐4 attenuates bleomycin‐induced lung fibrosis in mice. Purified recombinant wild‐type (wBP‐4) and IGFBP‐4 with a mutated IGF‐binding domain (mBP‐4) were intratracheally administered to mice (10 µg/dose for a total of three doses on days 0, 3, and 6). Mice were euthanized after 21 days. Histologic assessment of H&E‐stained sections shows that both wild‐type and mutant IGFBP‐4 reduced lung fibrosis. Images are representative of 3‐4 mice/group. (B) IGFBP‐4 reduces total collagen deposition as measured with a hydroxyproline assay. Mice were treated as in (A), and the left lung was used for measuring hydroxyproline levels. Both wBP4 and mBP4 significantly reduced hydroxyproline levels (number of mice/group is as indicated in the figure). (C) IGFBP‐4 reduces α‐SMA levels in bleomycin‐induced lung fibrosis in mice. Mice were treated as in A. The right lung was used for immunohistochemistry (left panel) and immunofluorescence (right panel) to detect α‐SMA, Hoechst was used to detect nuclei. *P < 0.05, **P < 0.01, ****P < 0.0001

Having demonstrated that IGFBP‐4 exerts antifibrotic effects in vitro and ex vivo, we tested its ability to ameliorate fibrosis in vivo in a murine bleomycin‐induced pulmonary fibrosis model. Wild‐type and mutant IGFBP‐4 proteins were generated as described in the methods and schematically shown in supplemental Figure S2A. We first confirmed the inability of IGFBP‐4 with a mutated IGF‐binding sequence to bind IGF‐I using western ligand blotting while wild‐type IGFBP‐4 retained its ability to bind IGF‐I (Figure S2B). Purified wild‐type and mutant rhIGFBP‐4 were intratracheally administered to mice that received a single dose of bleomycin. Mice received three doses of recombinant IGFBP‐4 on days 0, 3, and 6, and were euthanized 21 days post‐bleomycin administration. Histologic assessment of H&E‐stained sections shows that both wild‐type and mutant IGFBP‐4 reduced lung fibrosis (Figure 4A). The reduction in lung fibrosis was quantified using a hydroxyproline assay (Figure 4B) which showed that wild‐type and mutant IGFBP‐4 had similar antifibrotic effects and significantly reduced hydroxyproline content in mouse lungs. Furthermore, α‐SMA levels were also reduced following treatment with wild‐type and mutant IGFBP‐4 (Figure 4C). Since both wild–type and mutant IGFBP‐4 exerted comparable antifibrotic activity, we concluded that the antifibrotic effect of IGFBP‐4 is IGF independent.

### 3.5 IGFBP‐4 downregulates the SDF‐1 receptor CXCR4, and CXCR4 levels are elevated in primary pulmonary fibroblasts of patients with SSc‐associated pulmonary fibrosis

**Figure 5:**
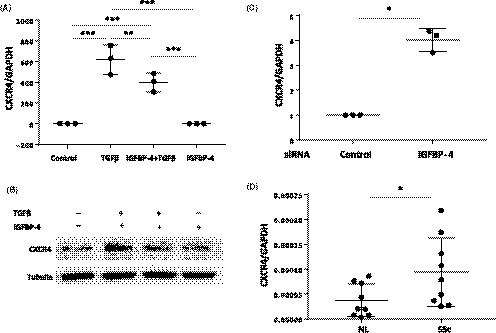
IGFBP‐4 downregulates CXCR4 and CXCR4 levels are elevated in SSc lung fibroblasts. (A) IGFBP‐4 inhibits baseline and TGF‐ß–induced CXCR4 mRNA expression. Primary human lung fibroblasts were treated with 2 µg/mL rhIGFBP‐4 or vehicle control with or without 10 ng/mL TGF‐ß1 for 24 hours. Total RNA was extracted and used for the measurement of CXCR4 mRNA levels by real‐time PCR. Data shown is from three independent experiments using fibroblasts from three different donors. (B) IGFBP‐4 reduces protein levels of CXCR4. Primary human lung fibroblasts were treated with 2 µg/mL rhIGFBP‐4 with or without 10 ng/mL TGF‐ß1 as described in (A). Cellular membrane fractions were analyzed by western blot. Experiments were repeated using lung fibroblasts from three different donors with similar results. (C) Silencing IGFBP‐4 increases CXCR4 expression. Primary human lung fibroblasts were transfected with siRNA targeting IGFBP‐4 or control scrambled siRNA for 24 hours. CXCR4 mRNA levels were measured by real time PCR. The graph summarizes results from three experiments using fibroblasts from three different donors. (D) CXCR4 mRNA levels are higher in SSc lung fibroblasts compared to normal control lung fibroblasts. CXCR4 mRNA levels were measured in fibroblasts from lung tissues of nine SSc patients and nine controls using real time PCR. *P < 0.05, **P < 0.01, ***P < 0.0001

To investigate the mechanisms by which IGFBP‐4 reduces fibrosis, we analyzed gene expression in human primary lung fibroblasts treated with IGFBP‐4 using PCR arrays (data not shown). We identified the SDF‐1 receptor CXCR4 as a gene whose expression is reduced by IGFBP‐4. These results were confirmed in unstimulated and TGF‐ß–stimulated primary normal human lung fibroblasts at both the mRNA (Figure 5A) and protein level (Figure 5B). Furthermore, silencing IGFBP‐4 increased the levels of CXCR4 mRNA in primary human lung fibroblasts (Figure 5C). Since several groups reported that the levels of CXCR4 were increased in SSc lung tissues, monocytes, and fibrocytes,^18^, ^19^, ^20^ we examined levels of CXCR4 in primary fibroblasts cultured from SSc lung tissues. Our results show that the levels of CXCR4 mRNA are significantly higher in SSc lung fibroblasts compared with control donor fibroblasts (Figure 5D). Since levels of IGFBP‐4 were significantly lower in SSc lung fibroblasts compared to control fibroblasts (Figure 3A) and IGFBP‐4 reduces CXCR4 while IGFBP‐4 deficiency increases CXCR4 levels (Figure 5A‐C), higher levels of CXCR4 in SSc patient lung fibroblasts may be due to reduced IGFBP‐4 levels.

### 3.6 IGFBP‐4 inhibits the expression of the profibrotic factor CTGF

**Figure 6:**
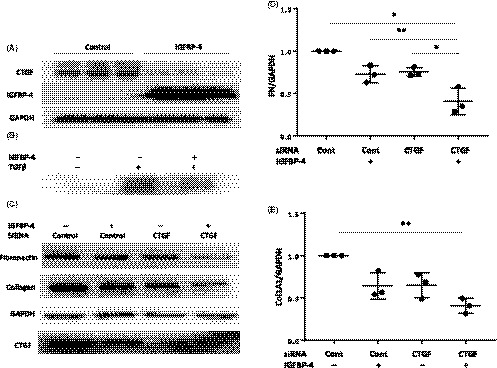
IGFBP‐4 reduces levels of the profibrotic factor CTGF. A. IGFBP‐4 reduces basal CTGF levels. Human primary lung fibroblasts were infected with replication‐deficient adenovirus encoding IGFBP‐4 or control adenovirus for 72 hours. CTGF protein levels were assessed in cellular lysates by WB. B. IGFBP‐4 inhibits TGF‐ß induction of CTGF. Primary human lung fibroblasts were treated with vehicle control or 10 ng/mL of TGF‐ß1 for 48 hours following 24 hours infection with replication‐deficient adenovirus encoding IGFBP‐4 or control adenovirus. Cell lysates were analyzed by WB. Experiments were repeated three times with similar results. C. Silencing CTGF potentiates the antifibrotic effect of IGFBP‐4. Human primary lung fibroblasts were transfected with siRNA targeting CTGF or control siRNA for 24 hours then treated with 2 µg/mL of rhIGFBP‐4 or vehicle for 48 hours. Cell lysates were analyzed by western blot. (D) Graphical presentation of densitometry results for FN from fibroblasts of three donors treated as in (C). (E) Graphical presentation of densitometry results for Col1A1 from fibroblasts of three donors treated as in (C). *P < 0.05, **P < 0.01

The expression of CTGF is increased in SSc fibroblasts.^21^, ^22^ Since IGFBP‐4 reduces baseline and TGF‐ß–induced ECM production and CTGF is a mediator of the profibrotic effects of TGF‐ß,^23^, ^24^ we examined the effect of IGFBP‐4 on CTGF. Endogenous expression of IGFBP‐4 in primary human lung fibroblasts inhibited baseline (Figure 6A) and TGF‐ß–induced (Figure 6B) CTGF levels. Furthermore, silencing CTGF potentiated the rhIGFBP‐4–induced reduction in the levels of the ECM proteins collagen 1A1 and fibronectin (Figure 6C‐E).

## 4 DISCUSSION

We demonstrate that IGFBP‐4 exerts antifibrotic effects as demonstrated in vitro using primary human pulmonary fibroblasts, in vivo using the murine bleomycin model of pulmonary fibrosis, and ex vivo using human skin and lung tissues in organ culture. Assays to delineate the mechanism(s) by which IGFBP‐4 reduces fibrosis identified downregulation of CXCR4, the SDF‐1 receptor responsible for trafficking of fibrocytes to the injured lung.^25^, ^26^ Additionally, IGFBP‐4 reduced levels of the profibrotic factor CTGF, which mediates the effects of TGF‐ß. These findings suggest that IGFBP‐4 may exert antifibrotic activity through more than one mechanism.

We previously reported that two other members of the IGFBP family of proteins, IGFBP‐3 and IGFBP‐5, are increased in pulmonary and dermal fibrosis and exert profibrotic effects,^4^, ^5^, ^7^, ^8^, ^9^, ^10^ in stark contrast to the effects of IGFBP‐4. This suggests that IGFBPs are pleiotropic and can exert opposite effects even in the same tissues. One can envision a scenario where the effects of IGFBP‐3 and IGFBP‐5 are counteracted by IGFBP‐4 in the setting of fibrosis. However, since we show that IGFBP‐4 levels are reduced in fibroblasts from patients with SSc‐associated pulmonary fibrosis, it is likely that an imbalance of IGFBPs can contribute to the development of fibrosis.

The function of IGFBP‐4 has been primarily explored in the setting of cell proliferation and angiogenesis, with a focus in cancer research. IGFBP‐4 inhibits IGF‐1 effects on cellular mitosis and proliferation.^27^, ^28^ IGFBP‐4 also plays a role as an antiangiogenic, antitumorigenic, cardiogenic growth factor, and inhibitor of canonical Wnt signaling functions that are IGF independent.^11^, ^29^ Furthermore, IGFBP‐4 has been shown to be one of several interferon‐beta (INF‐ß) response genes in primary B cells.^30^ These reports highlight the pleiotropic functions of IGFBP‐4 in multiple tissues and cellular processes.

Recent studies suggest that angiogenesis occurs early in injured tissues, followed by vascular apoptosis, and ultimately tissue fibrosis.^31^ In fact, in the bronchoalveolar lavage (BAL) fluid and lung tissues of patients with lung fibrosis, levels of angiogenic factors such as members of the CXC chemokines family (SDF/CXCR4), vascular endothelial growth factor (VEGF), platelet‐derived growth factor (PDGF), and others are increased and parallel increased levels of profibrotic factors such as TGF‐ß and IGF‐1.^32^, ^33^, ^34^, ^35^ Based on the known function of IGFBP‐4 in angiogenesis and our current work demonstrating its ability to ameliorate fibrosis, it is likely that IGFBP‐4 plays dual roles in different phases of the fibrotic response, modulating both angiogenesis and fibrosis. Examples of other antiangiogenic agents that also exhibit antifibrotic activity include endostatin and IFN‐gamma inducible protein 10.^17^, ^36^ Antiangiogenic factors may also modulate fibrosis via suppression of inflammation which often precedes fibrosis in SSc lung involvement. Our findings suggest that IGFBP‐4 has direct antifibrotic effects and reduces ECM and SMA expression. Thus, IGFBP‐4 may contribute to reduced fibrosis directly and indirectly via multiple mechanisms.

Our gene expression analysis in human primary lung fibroblasts treated with IGFBP‐4 showed that IGFBP‐4 significantly reduced CXCR4 and CTGF levels. This was confirmed with loss of function studies using sequence‐specific silencing of IGFBP‐4 expression. CXCR4 is a G‐protein coupled receptor that consists of seven transmembrane proteins and is widely expressed in various tissues. It is highly expressed in the hematopoietic and lymphatic systems and functions as a specific receptor for the chemokine SDF‐1. SDF‐1, acting via CXCR4, recruits circulating monocytes, fibrocytes, and other cells to the injured lung and their transformation into myofibroblasts, the effector cells in fibrosis.^2^ Over the past decade, small molecule CXCR4 inhibitors or antagonists were developed, including AMD3100 (Plerixafor), CTCE‐9908, and AMD 070. These antagonists were shown to ameliorate fibrosis in the murine bleomycin‐induced pulmonary fibrosis model^37^, ^38^, ^39^, ^40^ and in experimental burn scars,^41^, ^42^ partly as a result of reduced numbers of infiltrating inflammatory cells and decreased chemokine levels.^37^, ^38^, ^40^ Although one group did not detect a decrease in extracellular matrix deposition in injured lung, animal mortality was reduced as a result of a decreased inflammatory response.^43^

Our results demonstrate increased CXCR4 expression in SSc lung fibroblasts paralleling reduced levels of IGFBP‐4. The increased CXCR4 levels are consistent with the findings from other groups where CXCR4 expression was measured in lung tissues and peripheral blood monocytes.^18^, ^19^ A recent study reported that CXCR4 is the most commonly expressed marker in the differentiation of circulating progenitor cells in patients with SSc interstitial lung disease, followed by CD34 and CD45.^20^ We show that silencing IGFBP‐4 expression in human lung fibroblasts increased levels of CXCR4. Based on our findings, we propose that decreased levels of IGFBP‐4 in SSc fibroblasts may be responsible for increased CXCR4 in lung fibrosis and ultimately the promotion of CXCR4‐dependent chemotaxis of circulating monocytes and inflammatory cells, contributing to pulmonary fibrosis. The inflammatory microenvironment promotes increased production of SDF‐1 by macrophages and monocytes, resulting in recruitment of circulating fibrocytes to the injured lung via the SDF‐1 receptor, CXCR4, and subsequent differentiation of fibrocytes into myofibroblasts.^18^, ^44^ Blockade of SDF‐1/CXCR4 has been facilitated by the identification of small molecule CXCR4 inhibitors or antagonists under development as antifibrotic agents that significantly reduce pulmonary fibrosis in mice.^37^, ^38^, ^40^ In fact, SDF‐1 levels are increased in BAL fluid and levels of CXCR4 are increased in lung tissues in the bleomycin‐induced lung fibrosis mouse model.^37^, ^38^, ^39^ Increased CXCR4 has also been reported in SSc‐associated pulmonary fibrosis in lung tissues, circulating fibrocytes, and monocytes, as well as in lung tissues of patients with idiopathic pulmonary fibrosis (IPF).^18^, ^37^ The fact that IGFBP‐4 is upstream of CXCR4 and able to reduce its levels suggests that the antifibrotic effects of IGFBP‐4 are due, in part, to its modulation of CXCR4 levels, yet another novel function that has not been previously attributed to IGFBP‐4.

The antifibrotic activity of IGFBP‐4 was independent of MAPK, JNK, AKT, and p38 kinase signaling (data not shown). However, IGFBP‐4 decreased TGF‐ß–induced SMAD2/3 phosphorylation. TGF‐ß signaling is a central pathway in fibrosis.^45^ TGF‐ß interacts with its type I (TßRI) and type II (TßRII) receptors, resulting in the downstream phosphorylation of SMAD2 and SMAD3.^46^, ^47^ Phosphorylated SMAD2/3 translocate to the nucleus where they mediate the effects of TGF‐ß on gene transcription.^48^ C‐terminal domain phosphorylation of SMAD2/3 by TßRI kinase defines the TGF‐ß canonical signaling pathway,^49^ whereas phosphorylation of SMAD2 and SMAD3 linker domain by kinases such as MAPKs, ERK, and P38 is characteristic of the noncanonical TGF‐ß signaling pathway.^50^ Since the antibodies we used to detect SMAD2/3 phosphorylation detect the C‐terminal phosphorylation and since phosphorylation of p44/42 MAPK and p38 kinase was not observed following treatment of primary fibroblasts with IGFBP‐4 (data not shown), our findings suggest that IGFBP‐4 inhibits the canonical TGF‐ß signaling pathway. Inhibition of the canonical pathway has been shown to ameliorate pulmonary fibrosis. For example, sunitinib, a small molecule kinase inhibitor, reduced phosphorylation of serine residues on SMAD2/3 and attenuated bleomycin‐induced pulmonary fibrosis in mice.^51^

Our findings suggest that the antifibrotic activity of IGFBP‐4 is independent of IGF‐I since mutating the IGF binding domain of IGFBP‐4 did not abrogate the protein's antifibrotic effects in our models. The antifibrotic activity of IGFBP‐4 can be added to a growing list of other activities attributed to the IGF‐independent function of IGFBP‐4, such as its ability to reduce postoperative peritoneal adhesions in rats,^13^ its antiangiogenic and antitumorigenic effects, as well as its Wnt signaling inhibitory function,^11^, ^29^ some of which have been attributed to the carboxy‐terminal region of IGFBP‐4.^12^ IGFBP‐4 has also been reported to reduce postoperative peritoneal adhesions in rats via a mechanism that is believed to be IGF‐1 dependent.^13^ Thus, the IGF‐independent effects of IGFBP‐4 are emerging as important effects in multiple different processes in health and disease, rendering IGFBP‐4 an appealing target central to different disease processes. Whether the antifibrotic and antiangiogenic effects of IGFBP‐4 overlap in the setting of fibrosis remains to be explored. In fact, antiangiogenesis has been postulated to ameliorate fibrosis in different organs.^31^

Although TGF‐ß is considered a key mediator of fibrosis, other growth factors have also been shown to induce increased matrix production and deposition and organ fibrosis. It is thus unlikely that targeting a single gene will be sufficient for the treatment of human fibrosis as parallel profibrotic pathways are implicated. In that respect, use of IGFBP‐4 offers an attractive approach for reducing fibrosis as it modulates several different pathways including TGF‐ß signaling, levels of CXCR4, and production of CTGF.

## CONFLICT OF INTEREST

The authors have no relevant disclosures.

## AUTHOR CONTRIBUTIONS

YYS, TN, and CFB designed the research; JMP provided human lung tissues; YYS, TN, XXN, and SH performed the experiments; YYS and CFB analyzed the data and wrote the manuscript; all authors contributed to the editing of the manuscript.
