# Supplementary figures and images for: Insulin‐like growth factor binding protein‐4 exerts antifibrotic activity by reducing levels of connective tissue growth factor and the C‐X‐C chemokine receptor 4
Source: FASEB Bioadv. 2019 Jan 15;1(3):167–79. doi: 10.1096/fba.2018-00015 (PMC6720120; doi:10.1096/fba.2018-00015)

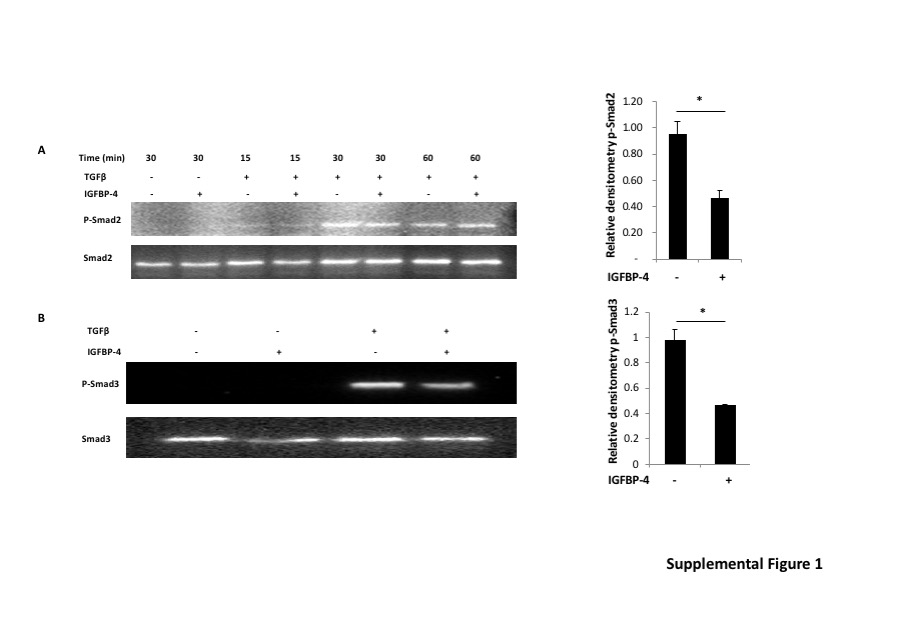

Supplement: Supplementary file 1 [file FBA2-1-167-s001.jpg]

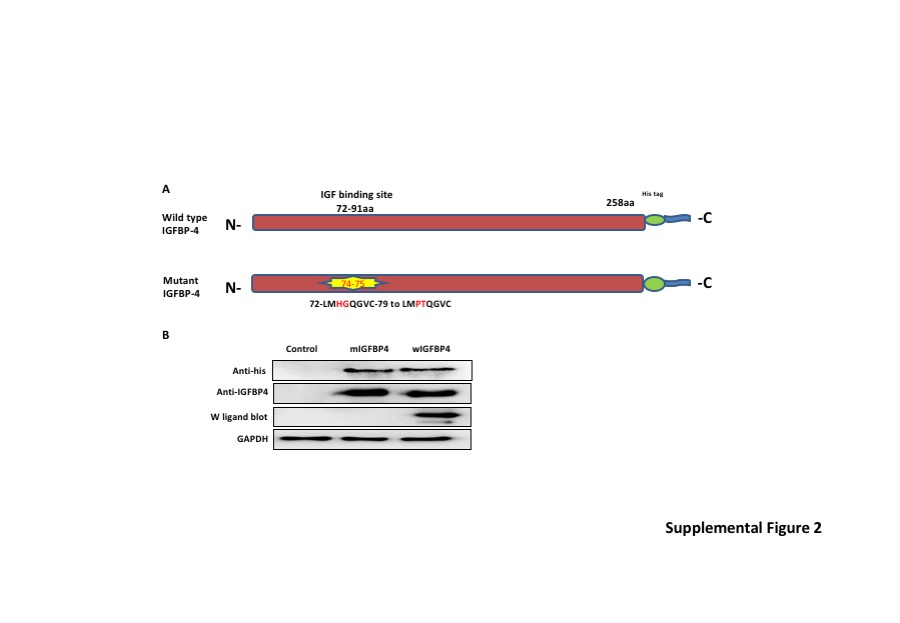

Supplement: Supplementary file 2 [file FBA2-1-167-s002.jpg]
