# Supplementary material for: Insulin‐like growth factor binding protein‐4 exerts antifibrotic activity by reducing levels of connective tissue growth factor and the C‐X‐C chemokine receptor 4
Source: FASEB Bioadv. 2019 Jan 15;1(3):167–79. doi: 10.1096/fba.2018-00015 (PMC6720120; doi:10.1096/fba.2018-00015)
Supplement: Supplementary file 3 [file FBA2-1-167-s003.pdf]

SUPPLEMENTAL FIGURE LEGENDS

Figure S1: IGFBP-4 reduces TGF-ß -induced SMAD 2/3 phosphorylation.

A. Human primary lung fibroblasts were treated in serum-free media with 2µg/ml human recombinant IGFBP-4 with or without 10ng/ml TGF-ß1 according for the indicated time points. Levels of phosphorylated and total Smad2 were assessed in cell lysates by western blot. IGFBP-4 reduced phosphorylation of SMAD2 30 minutes after TGF-ß stimulation. B. IGFBP-4 reduced phosphorylation of SMAD3 30 minutes after TGF-ß stimulation. The graphs show data from two independent experiments.

Figure S2: Generation of IGFBP-4-expressing constructs with 6x his tag.

A. Schematic of the cDNA of wild type IGFBP-4 and IGFBP-4 with a mutation in the IGF binding domain (mutant IGFBP-4) that were used to generate the adenoviral constructs. A 6x his tag was fused to the cDNA to facilitate recombinant protein purification. For mutant IGFBP-4, amino acids at positions 74 and 75 were mutated from histidine and glycine to proline and threonine, respectively. B. Western blot was used to confirm the expression of the wild type (wIGFBP4) and mutant (mIGFBP4) IGFBP-4. A western ligand blot was used to confirm the ability of wIGFBP-4 to bind IGF-1 and the inability of mIGFBP-4 to recognize IGF-1.
